# Quantitative integration of epigenomic variation and transcription factor binding using MAmotif toolkit identifies an important role of IRF2 as transcription activator at gene promoters

**DOI:** 10.1038/s41421-018-0045-y

**Published:** 2018-07-10

**Authors:** Hongduo Sun, Jiawei Wang, Zhaohui Gong, Jiaying Yao, Yuangao Wang, Jian Xu, Guo-cheng Yuan, Yijing Zhang, Zhen Shao

**Affiliations:** 1 CAS Key Laboratory of Computational Biology, CAS-MPG Partner Institute for Computational Biology, Shanghai Institute of Nutrition and Health, Shanghai Institutes for Biological Sciences, Chinese Academy of Sciences, Shanghai 200031, China; 2 University of Chinese Academy of Sciences, Beijing 100049, China; 3 National Key Laboratory of Plant Molecular Genetics, CAS Center for Excellence in Molecular Plant Sciences, Shanghai Institute of Plant Physiology and Ecology, Shanghai Institutes for Biological Sciences, Chinese Academy of Sciences, Shanghai 200032, China; 4 Children’s Medical Center Research Institute, Department of Pediatrics, University of Texas Southwestern Medical Center, Dallas, TX 75390 USA; 5 Department of Biostatistics and Computational Biology, Dana Farber Cancer Institute; Department of Biostatistics, Harvard T.H. Chan School of Public Health, Boston, MA 02115 USA; 6 Harvard Stem Cell Institute, Cambridge, MA 02138 USA

Dear Editor,

Eukaryotic gene transcription is controlled by a large cohort of chromatin-associated proteins including transcription factors (TFs) and epigenetic regulators^1,2^. ChIP-seq experiments are now widely used to characterize the genome-wide binding of these proteins, and comparing ChIP-seq data from different cell types can provide valuable insight into understanding how cell type-specific transcriptional programs are established^3^. Specifically, epigenetic regulators often show dynamic chromatin binding during development and disease progressions^2^. However, most of them are broadly expressed across tissues, and their chromatin binding is thought to be mainly modulated by crosstalk with TFs, which could be considered as their cell type-specific co-factors^4^. Thus, identifying TFs that preferentially bind at the genomic regions differentially bound by a chromatin-associated protein between different cell types has become an important step toward deciphering the molecular mechanism modulating its chromatin binding^4^. Moreover, applying this analysis to histone modifications marking active regulatory elements such as H3K4me1-3 and H3K27ac is frequently used for discovering cell type-specific regulators^5^.

A traditional way of this analysis is to first detect the cell type-specific ChIP-seq peaks of the protein of interest, which are typically defined as those that do not overlap with peaks identified from other cell types, and then search for TFs whose binding sites are significantly over-represented in these peaks^6^. But, the cell type-specific peaks defined in this way often suffer from high false-positive rates, which can severely affect the accuracy of downstream analysis^6,7^. Recently, it has been demonstrated that quantitative comparison of ChIP-seq data using MAnorm or other statistical models can more precisely characterize the differential binding of proteins than arbitrarily classifying their peaks into cell type-specific and non-specific ones based on peak overlap, and thus can provide a better basis for the following analysis^6–8^. This is particularly important for identifying the cell type-specific co-factors of the protein under study, which highly relies on both the sensitivity and specificity of the detection of differential binding^6^. Therefore, developing computational tools that systematically incorporate quantitative comparison of ChIP-seq data based on appropriate statistical models into the identification of cell-type specific regulators can effectively facilitate the application of these models.

Here, we present a practical toolkit, MAmotif, for this purpose. It can automatically perform quantitative comparison between ChIP-seq samples of the same protein but from different cell types, and identify TFs whose binding is significantly associated with the cell type-biased binding of this protein as its candidate co-factors (Fig. 1a). To assess its performance, we re-analyzed the ChIP-seq data of H3K4me3, a histone mark of active promoters, from adult and fetal human pro-erythroblast cells (proEs)^9^. More than 97% of the H3K4me3-associated genes (defined as genes with H3K4me3 peaks at promoters) are shared between adult and fetal stages, covering 93% of the genes differentially expressed between two stages (Supplementary Fig. S2a). However, using MAnorm model, we still identified hundreds of different H3K4me3 peaks at gene promoters, and the associated genes also tend to be differentially expressed (Supplementary Fig. S2b-d), indicating that the H3K4me3 levels at these genes are fine-tuned. Subsequently, we applied both MAmotif and traditional overlap-based approach to compare the ChIP-seq data. Interestingly, MAmotif identified IRF family motifs as the top candidate co-factors associated with adult-biased H3K4me3 peaks at gene promoters, while traditional overlap-based method ranked GATA2 motif as the most significant one (Fig. 1b). Of note, it has been validated that IRF2 can function as transcription activator at adult-specific enhancers^9^. Given that a significant fraction (19%) of IRF2 ChIP-seq peaks in adult proEs are located at gene promoters, we speculate IRF2’s promoter binding may also be important for adult proEs.

**Fig. 1:**
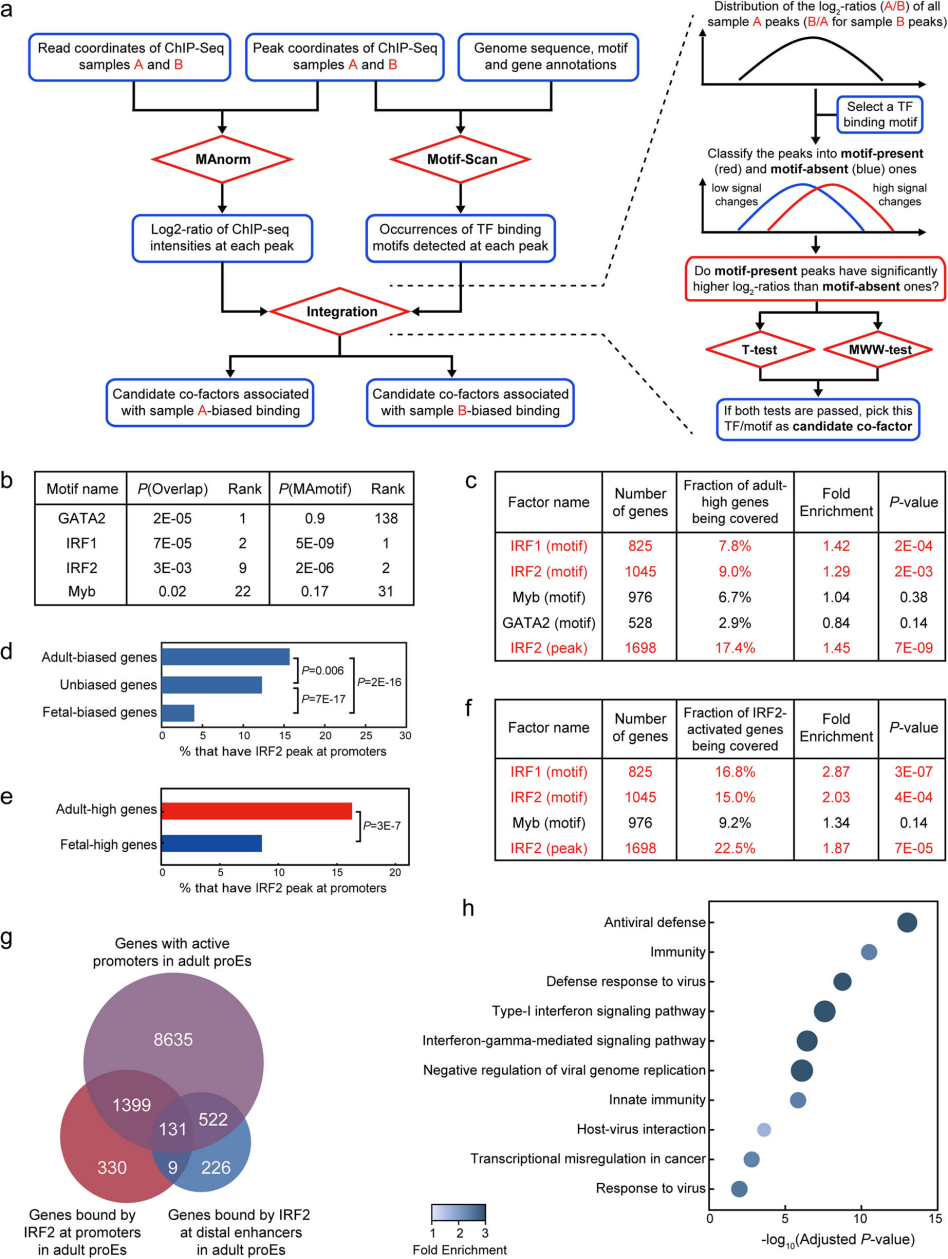
Using MAmotif to compare the H3K4me3 ChIP-seq data of adult and fetal proEs. **a** The overall workflow of MAmotif toolkit for comparing two ChIP-seq samples of the same chromatin-associated protein but from different cell types (a detailed introduction of the workflow and implementation of MAmotif toolkit and its Motif-Scan module can be found in Supplementary information and Supplementary Fig. S1b-f). Of note, MAmotif can also utilize TF binding information from other resources such as ChIP-seq data, instead of the TF binding motifs detected by its Motif-Scan module. **b** The top JASPAR motifs predicted by MAmotif and traditional overlap-based approach that are significantly associated with the adult-biased H3K4me3 promoter peaks compared to fetal proEs. **c** The overlap between adult-high genes and genes covered by the H3K4me3 promoter peaks of adult proEs that contain IRF1/2, MYB, GATA2 motifs, and IRF2 ChIP-seq peaks of adult proEs, respectively. **d** Fractions of adult-biased, fetal-biased, and unbiased H3K4me3-associated genes that have IRF2 peaks at their promoters. **e** Fractions of adult and fetal-high genes that have IRF2 peaks at promoters. Here the *P*-values shown in **d** and **e** were calculated by two-tailed Fisher’s exact test using hypergeometric distribution. **f** The overlap between IRF2-activated genes (genes downregulated after IRF2 knockdown in adult proEs) and genes covered by the H3K4me3 promoter peaks of adult proEs that contain IRF1/2, MYB motifs, and IRF2 ChIP-seq peaks, respectively. **g** Venn diagram shows the overlap between the genes with active promoters in adult proEs (covered by H3K4me3 peaks and not by H3K27me3 peaks, a repressive histone mark) and the IRF2 promoter/enhancer-bound genes of adult proEs. **h** Gene ontology (GO) enrichment analysis of the IRF2 promoter-bound genes in adult proEs. Here, all the active promoter genes of adult proEs were used as background and *P*-values were corrected by the Benjamini–Hochberg procedure for multiple testing

Next, we incorporated the gene expression profiles of adult and fetal proEs to test these predictions. The rationale is that if a TF does preferentially bind at the adult-biased H3K4me3 peaks, genes bound by it at promoters should be more likely to have adult-biased expression than other H3K4me3-associated genes, as H3K4me3 is a strong transcriptional activation mark^2^. By taking all 14,108 H3K4me3-associated genes of adult proEs as background, we found genes covered by the H3K4me3 promoter peaks containing IRF family motifs are significantly enriched in genes more highly expressed in adult proEs than fetal proEs (named as adult-high genes hereafter, Supplementary Fig. S2e and Fig. 1c) and depleted of genes more highly expressed in fetal proEs (named as fetal-high genes, Supplementary Fig. S2f). We repeated the analysis with IRF2 ChIP-seq peaks of adult proEs and observed a more significant enrichment (Fig. 1c–e). Moreover, we included the gene expression changes upon shRNA-mediated knockdown of IRF2 in adult proEs^9^. Strikingly, a significantly higher fraction of the genes downregulated after IRF2 knockdown (named as IRF2-activated genes) were covered by the H3K4me3 promoter peaks co-occupied by IRF2 than expected by chance (Fig. 1f), indicating that IRF2’s promoter binding is linked with transcriptional activation of downstream genes. On the other hand, the presence of GATA2 motif at H3K4me3 promoter peaks failed to show any significant association with adult-biased gene expression (Fig. 1c). Then, we defined stage-biased H3K4me3 peaks based on the log_2_-ratios of H3K4me3 intensities and corresponding *P*-values, and confirmed that the IRF2 motif, but not GATA2 motif, is significantly enriched in adult-biased H3K4me3 promoter peaks compared to the fetal-biased ones (Supplementary Fig. S2g-h), especially in those adult-biased peaks co-localized with IRF2 peaks (Supplementary Fig. S2i). This is consistent with the previous finding that GATA TFs regulate erythropoiesis at both stages^10^.

We have shown that besides distal enhancers, IRF2 can also function as transcription activator at gene promoters. However, only a small fraction of IRF2 promoter-bound genes overlap with the genes associated with IRF2-bound enhancers (Fig. 1g), though the vast majority of these genes have active promoters in adult proEs (marked by H3K4me3 but not by repressive mark H3K27me3). Next, by using all the active promoter genes as background, we confirmed IRF2’s binding at promoter and enhancer regions is regulating different pathways in adult proEs. More specifically, the IRF2 promoter-bound genes are highly enriched in immune and viral response pathways (Fig. 1h), while genes bound by IRF2 at enhancers are more closely related with basic cellular functions such as RNA transcription and protein phosphorylation (Supplementary Fig. S2j). It should be noted that immune pathways comprise one of the key differences between adult and fetal proEs at transcriptome level, but the molecular mechanism has not yet been deciphered^9^. With our new analysis, now it is clear that IRF2 preferentially regulates a considerable number of immune-related genes by directly binding at their promoters, and these genes tend to be more highly expressed in adult proEs (Supplementary Fig. S2k), suggesting that it plays an important role of transcriptional activation at promoters.

In summary, we present a new computational toolkit, MAmotif, for detecting co-factors associated with the differential chromatin binding of proteins, based on quantitative comparison of their ChIP-seq data and systematic integration with TF binding information from motif analysis or other resources. Applying it to real ChIP-seq data, we unveiled an important role of IRF2 as transcription activator at gene promoters through coupling with H3K4me3 mark, which clearly illustrates the power of quantitative integration of epigenomic variation and TF binding information at regulatory elements using MAmotif for detecting cell type-specific regulators. MAmotif toolkit is available at https://github.com/shao-lab/.

## Electronic supplementary material

Supplementary Information
