# Supplementary material for: Quantitative integration of epigenomic variation and transcription factor binding using MAmotif toolkit identifies an important role of IRF2 as transcription activator at gene promoters
Source: Cell Discov. 2018 Jul 10;4:38. doi: 10.1038/s41421-018-0045-y (PMC6037672; doi:10.1038/s41421-018-0045-y)
Supplement: Supplementary file 1 — Supplementary Information [file 41421_2018_45_MOESM1_ESM.pdf]

Quantitative integration of epigenomic variation and transcription factor binding using MAmotif toolkit identifies an important role of IRF2 as transcription activator at gene promoters

Supplementary information

Public data and web tools used in this study

The raw ChIP-seq data and detected peaks of histone modification H3K27ac and related TFs in H1 human embryonic stem cells (hESCs), K562 human leukemia cells and human embryonic kidney HEK293 cells were obtained from ENCODE website (http://genome.ucsc.edu/ENCODE/). In this study, we used the position weight matrixes (PWMs) of all the 198 vertebrate TF binding motifs downloaded from JASPAR database in 2014 [1]. ChIP-seq data of histone modification H3K4me3 and TF IRF2 in adult and fetal proEs as well as the gene expression data were obtained from Xu et al [2]. From Cistrome Data Browser website, 6092 sets of human TF binding peaks from public ChIP-seq data were downloaded [3]. Gene ontology (GO) analysis was performed using DAVID website with default parameters [4]. RefSeq gene annotations of human genome assembly hg18 were downloaded from UCSC genome browser, and the promoter of each gene was defined as the region from 2 kb upstream to 2 kb downstream of its annotated transcriptional start site (TSS). The Fisher's exact tests were performed using R software to calculate *P*-values based the hypergeometric distribution, and fold enrichment of the overlap between two gene sets was defined as the ratio between the number of overlapped genes and that expected by chance.

MAmotif uses quantitative comparison of ChIP-seq data to detect co-factors associated with the differential binding

MAmotif is used to quantitatively compare two ChIP-seq samples of the same protein but from different cell types (or conditions) and identify co-factors associated with the cell type-biased binding of this protein using the binding information of candidate TFs obtained from motif analysis (or other resources such as ChIP-chip/seq data). In MAmotif, we switch to use the $\log_2$-ratio of ChIP-seq intensities between two samples at each peak, i.e. the **M** value calculated by MAnorm model [5], to quantitatively represent the cell type specificity of this peak. This is mainly due to two reasons. First, when comparing ChIP-seq samples from different cell types, it was frequently found that the cell type specificity of peaks cannot be precisely characterized by simply classifying them into cell type-specific and non-specific ones by whether or not they overlap with peaks identified from other cell types [5-7], especially when the ChIP-seq samples are from two closely related cell types or conditions [2]. On this account, it has been suggested to better describe the binding changes in a quantitative way [5, 7]. Second, it was also found that genomic regions with greater chromatin state changes across cell types usually are more likely to be directly co-occupied by key cell type-specific regulators [8]. As an example, we applied MAnorm to compare the ChIP-seq data of H3K27ac, a histone mark of active gene promoters and distal enhancers, between H1 hESCs and K562 cells. After ranking all the H3K27ac peaks by their log2-ratios of ChIP-seq intensities between two cell types, it can be clearly viewed that peaks with higher hESC-biased H3K27ac levels are more likely to contain ChIP-seq peaks of hESC-specific regulators POU5F1, SOX2 and NANOG, while peaks with higher K562-biased H3K27ac levels are more likely to be co-occupied by K562-specific regulators TAL1 and GATA1 (Supplementary Fig. S1a). To test whether we can reproduce this observation with motif analysis, we scanned all the H3K27ac peaks of two cell types with the vertebrate TF binding motifs downloaded from JASPAR database [1] using our Motif-Scan toolkit (a detailed description of this toolkit can be found in the following sections), which has been successfully tested in our previous studies [2, 5, 9-11]. Consistently, we found H3K27ac peaks with greater ChIP-seq intensity changes showed a higher enrichment of corresponding cell type-specific regulators'

binding motifs (Supplementary Fig. S1e), which in turn could also support the effectiveness of Motif-Scan toolkit in predicting TF binding sites. On the other hand, for each motif, we systematically classified the H3K27ac peaks identified from each cell type into motif-present and motif-absent ones, based on whether or not they were detected to contain this motif in their sequences by Motif-Scan. Interestingly, it could be easily found that peaks having the binding motifs of POU5F1, SOX2, TAL1 and GATA1 exhibited obviously greater changes of H3K27ac levels than the corresponding motif-absent peaks (Supplementary Fig. S1f). Meanwhile, peaks with the binding motifs of SPZ1 and HNF4G, two TFs that haven't been reported to have clear functional selectivity between two cell types, failed to show significantly different levels of H3K27ac changes compared to the other peaks (Supplementary Fig. S1f). Taken together, these analyses not only validated the power of quantitative comparison on ChIP-seq data, but also shed light on how to utilize the quantitative measure of ChIP-seq intensity changes.

Based on these observations, we combine MAnorm and Motif-scan as two basic functional modules of MAmotif, and incorporate a new integration module to examine the association between the quantitative changes of ChIP-seq intensities calculated by MAnorm and the occurrences of each TF binding motif detected by Motif-Scan. The significance of this association could be used to infer whether the corresponding TF can be a candidate cell type-specific co-factor accounting for the cell type-biased binding detected between the two ChIP-seq samples under comparison. Here we briefly describe its workflow (Figure 1). First, it takes the coordinates of the peaks and aligned reads of two ChIP-seq samples as main input, together with the TF binding motifs chosen for motif analysis. Then, the input data are automatically processed by MAnorm and Motif-Scan modules, to get the log2-ratio of normalized ChIP-seq read densities as well as the occurrences of all input motifs at each peak region. Subsequently, they are sent to the integration module, and two statistical tests are applied to assess the association between the ChIP-seq intensity changes and the occurrence of each input motif (right panel of Figure 1a), by checking whether the peaks having this motif (named as

motif-present peaks) show significantly higher ChIP-seq intensity changes between two ChIP-seq samples than peaks with no motif occurrence (named as motif-absent peaks). If a motif passes both tests, which means the motif-present peaks of this motif are significantly more likely to have cell type-biased binding than the motif-absent peaks, this motif (i.e. the corresponding TF) will be reported as a candidate cell type-specific co-factor.

Introduction of the workflow and implementation of MAmotif toolkit

MAmotif takes four bed files describing the coordinates of all predefined peaks and aligned reads of two ChIP-seq samples as the main input. Besides, it also needs the gene annotation file of the corresponding genome assembly and fasta files of genome sequences, as well as a text file containing the PWMs of all candidate motifs as input for motif analysis. Of note, users can use the processed motif data for the motifs in JASPAR database (including the PWM of each motif and the motif score cutoffs corresponding to difference significance levels), which can be found on the webpage of Motif-Scan, to directly run a MAmotif analysis.

MAmotif starts from processing the input peak and read information of two ChIP-seq samples using MAnorm module to calculate the log2-ratio of normalized ChIP-seq intensities (represented by the ChIP-seq read densities) at each peak region (extended/truncated to the same length from peak summit, and here this length is set to be 2kb for H3K27ac and H3K4me3 as suggested for histone modifications with sharp peaks). Meanwhile, the DNA sequence of each peak region is extracted and scanned by the Motif-Scan module with input motifs, to detect the occurrence of each candidate motif in the sequence with motif score higher than the cutoff specified by the user. Here the length of peak region for motif scanning is set to be 1 kb as suggested. Next, the log2-ratios of ChIP-seq intensities calculated by MAnorm and the occurrences of each candidate motif detected by Motif-Scan are combined in the integration module. For each motif, all the peaks identified from one cell type, e.g. cell type A, are systematically classified into motif-present and motif-absent ones, based on whether or not it is

found to contain at least one occurrence of this motif. Here users can choose to exclude the motifs being present in a very large fraction of peaks (e.g. >50%), which usually are motifs with low information content and may introduce a lot of false positives. Then, two statistical tests, including the Students' t-test and Mann–Whitney–Wilcoxon (MWW) rank-sum test, are applied to test whether the motif-present peaks generally have higher log2-ratios of ChIP-seq intensities (the ratios are calculated as cell type A/B) than motif-absent ones. Finally, the P-values of the two tests are corrected for multiple testing using the Benjamini–Hochberg approach, and the less significant one is used to rank the candidate motifs. Of note, MAmotif provides an option to separate promoter and non-promoter peaks based on input gene annotations before doing the statistical tests, since it was often found that the cell type-biased binding of many chromatin-associated proteins, especially the histone modifications, may be associated with different co-factors at gene promoter and distal regions. Besides, MAmotif can also be used to compared DNase-seq and ATAC-seq data and identify candidate TFs associated with the cell type-biased open chromatin sites.

Currently MAmotif is written in Python. The stand-alone version of all its three main functional modules are also provided. Their source codes and user manuals can be found at https://github.com/shao-lab/ and http://bioinfo.sibs.ac.cn/shaolab/opendata.php.

Workflow of the Motif-Scan toolkit embedded in MAmotif

Motif-Scan is a computational toolkit designed to scan input genomic regions with known DNA motifs, and check whether any of the motifs are significantly over- or under-represented in input genomic regions compared to the control regions randomly selected from the genome (Supplementary Fig. S1b), or compared to another set of genomic regions provided by the user as controls (this option can be used to identify motifs differentially enriched in two groups of genomic regions, e.g. the unique peaks identified from two ChIP/DNase/ATAC-seq samples). Motif-Scan takes a set of genomic regions (or two sets of genomic regions to perform differential

enrichment analysis), the DNA motifs of interest, as well as the fasta file of each individual chromosome's DNA sequence and the gene annotation file of the corresponding genome assembly as input. If the input genomic regions are ChIP/DNase/ATAC-seq peaks, we suggest to truncate/extend them to the same length (from peak center by default, or from peak summit if available) before doing motif scanning, in order to make the number of a motif's occurrences comparable across different peaks. Here the length for motif scanning can be customized by the users (otherwise they can choose to scan the entire input regions without any truncation/extension), which is suggested to be 1 kb for DNase/ATAC-seq peaks and ChIP-seq peaks of TFs and histone modifications with sharp peaks. If the user did not provide any control regions, Motif-Scan will select a set of random control regions from the genome for each input region (typically the number is set to be 5), with controlling the distance to the nearest gene's TSS to be the same as the input region (unless it is far from known genes' TSSs, e.g. with a distance to the nearest gene's TSS >10kb). In this way, the random control regions can be assumed to have a similar sequence background to the input regions, as gene promoters often have high GC content and are rich of TF binding motifs.

After extracting the genome sequences of all the input and control regions, for each motif Motif-Scan uses a sliding window with the same length of the motif to scan the sequences at a step size of 1 base pair (bp). At each step, a motif score is calculated to represent the similarity between the sequence fragment in the window and the motif (Supplementary Fig. S1c), which equals to 1 if the sequence fragment is right the best match of the motif, given the genome background nucleotide frequencies [5, 10]. Then, the motif score is compared with the genome background motif score distribution of this motif, which is generated from $5*10^6$ sequence fragments randomly sampled from the genome, and the sequence fragment will be labeled as a significant match of the motif if its motif score is higher than the cutoff specified by user. Here we suggest to use the motif score cutoff corresponding to a certain significance level, e.g. *P*-value 0.0001 based on the genome background motif score distribution. It means that among 10000

random sequence fragments, it's expected to have at most one sequence fragment with motif score higher than this cutoff. Besides providing the users with a script to model the background motif score distribution in a specific genome with their own motifs, we also compiled the motif score cutoffs corresponding to difference significance levels for the TF-binding motifs obtained from JASPAR database [1, 12] in several frequently used genomes (such as human and mouse). In this way, the users can use these motif annotation files to directly perform motif scanning with all JASPAR motifs.

When motif scanning is done, several statistics will be calculated for each motif to represent its enrichment/depletion in input genomic regions as compared to the control regions: 1) fold enrichment calculated as the ratio between the fraction of input regions having this motif and that of control regions, with the value higher/lower than 1 indicating the motif is over/under-represented (enriched/depleted) in input regions, respectively; 2) a P-value calculated based on hyper-geometric distribution to represent whether the enrichment/depletion is of statistical significance.

Since Motif-Scan is mainly developed for performing motif analysis on peaks identified from ChIP/DNase/ATAC-seq data, it will additionally output several figures to visualize the enrichment and also the distribution of motif occurrences in the input regions (Supplementary Fig. S1d). They can help users to assess the association between a motif's occurrence and the ChIP/DNase/ATAC-seq signal intensities at peak regions. This analysis can be further used to infer the data quality if the association is well established. For example, the occurrence of a TF's binding motif typically is positively correlated with the ChIP-Seq intensities at its ChIP-Seq peaks. More specifically, peaks with stronger ChIP-seq intensities are more likely to contain its binding motifs (as shown in the left panels of Supplementary Fig. S1d), and within each peak, its binding motif is also more likely to be observed at the peak summit compared to flanking regions (as shown in the right panels of Supplementary Fig. S1d).

MAmotif can also utilize TF binding information from available ChIP-chip/seq data

Inspired by the analysis with IRF2 ChIP-seq peaks, we additionally provide a function in MAmotif toolkit to utilize TF binding information obtained from other resources, such as peaks of ChIP-chip/seq samples, to test the association between each candidate TF's binding and the ChIP-seq signal changes detected from the samples under comparison. To validate the effectiveness of this approach, we repeated the MAmotif analysis with all the human TF ChIP-seq peaks downloaded from Cistrome Data Browser [3], instead of the occurrences of JASPAR motifs detected by Motif-Scan. Consistently, we found only the IRF2 ChIP-seq peaks of adult proEs exhibited significant association with adult-biased H3K4me3 promoter peaks (*P*-value=3e-5). This finding not only validates the reproducibility of our prediction based on motif analysis, but also suggests this additional function of MAmotif can provide a valuable approach to reuse the huge amount of public ChIP-seq data.

Traditional overlap-based approach used in this study to detect co-factors associated the differential H3K4me3 peaks between adult and fetal proEs

In this study, the traditional overlap-based approach was also used to compare the H3K4me3 ChIP-seq data of adult and fetal proEs and identify co-factors associated with adult-specific H3K4me3 promoter peaks. First, adult-specific H3K4me3 peaks were defined as the H3K4me3 peaks of adult proEs that do not overlap with any H3K4me3 peak detected in fetal proEs, and the other H3K4me3 peaks of adult proEs were named as non-specific peaks. Then, Motif-Scan was applied to scan the adult-specific and non-specific H3K4me3 promoter peaks with the same parameters as those used in MAmotif analysis, and a *P*-value was calculated for each motif based on hypergeometric distribution to represent its differential enrichment between adult-specific H3K4me3 promoter peaks and the non-specific peaks, which was used to finally select the top candidate motifs.

Analysis of IRF2's binding at the differential H3K4me3 peaks defined by MAnorm

To more clearly illustrate the association between IRF2's binding and the H3K4me3 promoter peaks with stage-biased H3K4me3 levels, here we further defined stage-biased H3K4me3 peaks based on the quantitative comparison of H3K4me3 ChIP-seq data between adult and fetal proEs using MAnorm. Here, adult and fetal-biased H3K4me3 peaks were defined as the H3K4me3 peaks identified from adult and fetal proEs with fold change of H3K4me3 ChIP-seq intensities higher than 1.5 between adult and fetal proEs and *P*-value lower than 0.05, and unbiased H3K4me3 peaks were defined as the H3K4me3 peaks with fold change of H3K4me3 ChIP-seq intensities lower than 1.5. It can be seen that a significantly higher fraction of adult-biased H3K4me3 promoter peaks were detected to contain IRF2 motifs by Motif-Scan compared to fetal-biased and unbiased H3K4me3 promoter peaks (Supplementary Fig. S2g), while the GATA2 motif was not found to be significantly more enriched in adult-biased H3K4me3 promoter peaks compared to fetal-biased ones (Supplementary Fig. S2h). These findings can directly support the predictions by MAmotif as shown in Figure 1b. Then, we named the adult-biased H3K4me3 promoter peaks that are detected to contain IRF2 motifs as IRF2-motif-present adult-biased H3K4me3 promoter peaks, and the others as IRF2-motif-absent adult-biased H3K4me3 promoter peaks. It could be found that the IRF2-motif-present adult-biased H3K4me3 promoter peaks are much more likely to directly co-localize with the IRF2 ChIP-seq peaks of adult proEs than IRF2-motif-absent adult-biased H3K4me3 promoter peaks (Supplementary Fig. S2i), suggesting IRF2's binding at adult-biased H3K4me3 promoter peaks is largely mediated by it's known binding motif.

Next, we defined the adult and fetal-biased H3K4me3-associated genes as genes with adult and fetal-biased H3K4me3 peaks at promoters, respectively, and the unbiased H3K4me3-associated genes as genes that only have unbiased H3K4me3 peaks at their promoters. Consistently, we found a much higher fraction of adult-biased H3K4me3-associated genes are directly bound by IRF2 at promoters in adult proEs than that of fetal-biased

H3K4me3-associated genes (Figure 1d). Besides, the adult-high genes are also found to be more likely to have IRF2 ChIP-seq peaks at their promoters in adult proEs compared to those fetal-high genes. These findings clearly indicate that IRF2 preferentially binds at the promoters of genes with adult biased promoter H3K4me3 and expression levels, as predicted by MAmotif.

Association between IRF2's binding and other histone modifications at gene promoters

We additionally checked the association between IRF2 and adult-biased H3K4me1 and H3K27ac peaks at gene promoters. First, we applied MAnorm to re-analyze the ChIP-seq data of H3K4me1 and H3K27ac in adult and fetal proEs, and defined the adult/fetal-biased H3K4me1 and H3K27ac peaks as those with fold change of ChIP-seq intensities higher than 1.5 and P-value lower than 0.05. Interestingly, the fraction of adult-biased H3K27ac and H3K4me1 promoter peaks with the presence of IRF2 motif was not found to be significantly higher than that of other H3K27ac and H3K4me1 peaks (Supplementary Fig. S3a-b). Consistently, the fraction of adult-biased H3K27ac/H3K4me1-associated genes co-occupied by IRF2 ChIP-Seq peaks at promoters were also not found to be significantly higher than that of other H3K27ac/H3K4me1-associated genes (Supplementary Fig. S3c-d). Besides, we also tried mapping the IRF2 peaks located at annotated gene promoters to the second nearest genes of them, and found these genes showed a much weaker enrichment in the adult-high genes (Supplementary Fig. S3e). Especially, these genes failed to show any significant enrichment in the IRF2-activated genes (Supplementary Fig. S3f), suggesting IRF2's promoter binding mainly regulates the activity of immediate downstream genes.

Discussion of the performance of different approaches in identifying differential peaks between ChIP-seq samples and detecting co-factors associated with differential binding

In the comparison of H3K4me3 ChIP-seq data between adult and fetal proEs, 97% of the H3K4me3-associated genes are shared between two stages, covering more than 90% of the

differentially expressed genes (Supplementary Fig. S2a). However, by using MAnorm to perform a quantitative comparison of ChIP-seq data, we still found many genes have stage-biased H3K4me3 levels at their promoters, and the changes of their H3K4me3 levels obviously are correlated with their expression changes (Supplementary Fig. S2b-d). Thus, it's reasonable to find that traditional overlap-based analysis did not achieve a plausible performance in identifying co-factors associated with the differential H3K4me3 promoter peaks, as most of the real differential peaks were actually missed by this approach.

But, this finding does not mean the traditional overlap-based approach is useless. When applied to compare more distinct cell types, it may have an improved performance. Here we again use the comparison of H3K27ac ChIP-seq data between H1 hESCs and K562 cells as an example. The majority of the H3K27ac peaks of two cell types were labeled as cell type-specific ones based on peak overlap [5], leading to a large number of cell type-specific H3K27ac-associated genes (genes having H3K27ac peaks at their promoters). These genes cover a substantial part of the genes differentially expressed between two cell types (Supplementary Fig. S4a). After using MAnorm to compare the two ChIP-seq samples, it can be easily seen that both the promoter H3K27ac level and the expression level of the cell type-specific H3K27ac-associated genes change dramatically between two cell types (Supplementary Fig. S4c). Then, we applied both traditional overlap-based approach and MAmotif to identify the co-factors associated with H1-specific/biased H3K27ac peaks. Interestingly, the top 5 JASPAR motifs detected by traditional overlap-based approach are exactly the same as those predicted by MAmotif (some of them may have different ranks, Supplementary Fig. S4d), which cover several known pluripotency related TFs including Pou5f1, TCF3 and Sox family motifs (its family member Sox2 is widely known as a core pluripotency TF). This finding indicates that when most of the cell type-specific peaks (or associated genes) defined by overlapping analysis are true differential peaks, they can still be used as the basis for the following analysis (but as suggested in our previous studies [2, 5], we recommend to use a

quantitative/statistical comparison of the corresponding ChIP-seq samples to validate the definition of differential and non-differential peaks from overlapping analysis and/or further filter out those unreliable ones, in order to achieve a better performance).

In some recent studies, DESeq and DESeq2 [13, 14], which were originally developed for differential expression analysis of RNA-seq data, were also used to perform differential binding analysis with ChIP-seq data of histone modifications. Here we also tried using DEseq2 and MEME [15], a very famous motif analysis suite, to re-perform the comparison of H3K4me3 ChIP-seq data between adult and fetal proEs. First, we found DEseq2 failed to detect any H3K4me3 peak with significant ChIP-seq intensity change using $P<0.01$ as cutoff (Supplementary Fig. S5b), while MAnorm can still detect more than two thousand differential H3K4me3 peaks with the same cutoff, and several hundred of them are located at gene promoters, covering a considerable fraction of genes differentially expressed between adult and fetal proEs (Supplementary Fig. S5b). Then, we switched to use a less stringent cutoff *P*-value 0.05 as cutoff, and obtained 86/119 adult/fetal-biased H3K4me3 promoter peaks from DEseq2, respectively. We applied the AME tool in MEME suite to analyze the relative enrichment of the 196 JASPAR vertebrate motifs used in our study between the adult and fetal-biased H3K4me3 promoter peaks detected by DEseq2, and none of these motifs were found to be significantly more enriched in adult-biased H3K4me3 promoter peaks compared to the fetal-biased ones ($P<0.01$ after correction for multiple testing). On the other hand, AME found IRF family motifs are significantly over-represented in the adult-biased H3K4me3 promoter peaks detected by MAnorm compared to the corresponding fetal-biased peaks ($P=2E-7$ for IRF2 motif), indicating AME has the ability to detect motifs truly associated with adult-biased H3K4me3 peaks and our finding can be reproduced by other motif analysis tools.

Of note, the developers of DEseq and DEseq2 have claimed they were originally designed to compare RNA-seq data with biological replicates, so it’s not too surprising to find they do not have a good performance on the ChIP-seq data used in this study. Besides, it has been

mentioned that it's better to use computational tools specifically designed for ChIP-seq data to perform differential binding analysis [16]. For example, the signal-to-noise (S/N) ratio can vary dramatically across ChIP-seq samples, and it could be an important issue for the comparison of ChIP-seq data. Here, we still use the comparison of H3K27ac ChIP-seq data between H1 hESCs and K562 cells as an example. We applied both DEseq2 and MAnorm to compare the H3K27ac ChIP-seq data, and found DEseq identified significantly more H1-biased H3K27ac peaks than K562-biased ones (2669 vs 357, Supplementary Fig. S5e) using fold change higher than 2 and *P*-value lower than 0.01 as cutoffs, while the numbers of the H1-biased and K562-biased H3K27ac peaks detected by MAnorm are still quite comparable with each other (15572 vs 13562, Supplementary Fig. S5e), covering a much higher proportion of the genes differentially expressed between H1 and K562 cells (38.6% and 54.1% of H1 and K562-high genes, respectively, compared to 0.7% and 0.1% by DEseq2, Supplementary Fig. S5e). Interestingly, we previously found that DEseq2 detected similar numbers of adult and fetal-biased H3K4me3 peaks between adult and fetal proEs. Then, we used the fraction of aligned reads covered by the top 15,000 peaks as a simple estimation of the S/N ratio of each ChIP-seq sample, and found the H3K27ac ChIP-seq samples of H1 and K562 cells do have quite different S/N ratios, while the H3K4me3 samples of adult and fetal proEs showed similar S/N ratios with each other (Supplementary Fig. S5f), indicating the unbalanced numbers of the differential peaks detected by DEseq are very likely to be due to the variation of S/N ratio between these samples.

References

1. Sandelin, A., et al., *JASPAR: an open-access database for eukaryotic transcription factor binding profiles.* Nucleic Acids Res, 2004. **32**(Database issue): p. D91-4.
2. Xu, J., et al., *Combinatorial assembly of developmental stage-specific enhancers controls gene expression programs during human erythropoiesis.* Dev Cell, 2012. **23**(4): p. 796-811.
3. Mei, S., et al., *Cistrome Data Browser: a data portal for ChIP-Seq and chromatin accessibility data in human and mouse.* Nucleic Acids Res, 2017. **45**(D1): p. D658-D662.
4. Huang da, W., B.T. Sherman, and R.A. Lempicki, *Systematic and integrative analysis of large gene lists using DAVID bioinformatics resources.* Nat Protoc, 2009. **4**(1): p. 44-57.

5. Shao, Z., et al., *MAnorm: a robust model for quantitative comparison of ChIP-Seq data sets.* Genome Biol, 2012. **13**(3): p. R16.
6. Xu, H., et al., *An HMM approach to genome-wide identification of differential histone modification sites from ChIP-seq data.* Bioinformatics, 2008. **24**(20): p. 2344-9.
7. Bailey, T., et al., *Practical guidelines for the comprehensive analysis of ChIP-seq data.* PLoS Comput Biol, 2013. **9**(11): p. e1003326.
8. He, H.H., et al., *Differential DNase I hypersensitivity reveals factor-dependent chromatin dynamics.* Genome Res, 2012. **22**(6): p. 1015-25.
9. Baena, E., et al., *ETV1 directs androgen metabolism and confers aggressive prostate cancer in targeted mice and patients.* Genes Dev, 2013. **27**(6): p. 683-98.
10. Liu, Y., Z. Shao, and G.C. Yuan, *Prediction of Polycomb target genes in mouse embryonic stem cells.* Genomics, 2010. **96**(1): p. 17-26.
11. Huang, J., et al., *Dynamic Control of Enhancer Repertoires Drives Lineage and Stage-Specific Transcription during Hematopoiesis.* Dev Cell, 2016. **36**(1): p. 9-23.
12. Matys, V., et al., *TRANSFAC: transcriptional regulation, from patterns to profiles.* Nucleic Acids Res, 2003. **31**(1): p. 374-8.
13. Love, M.I., W. Huber, and S. Anders, *Moderated estimation of fold change and dispersion for RNA-seq data with DESeq2.* Genome Biol, 2014. **15**(12): p. 550.
14. Anders, S. and W. Huber, *Differential expression analysis for sequence count data.* Genome Biol, 2010. **11**(10): p. R106.
15. Bailey, T.L., et al., *MEME SUITE: tools for motif discovery and searching.* Nucleic Acids Res, 2009. **37**(Web Server issue): p. W202-8.
16. Nakato, R. and K. Shirahige, *Recent advances in ChIP-seq analysis: from quality management to whole-genome annotation.* Brief Bioinform, 2017. **18**(2): p. 279-290.

Supplementary Figures

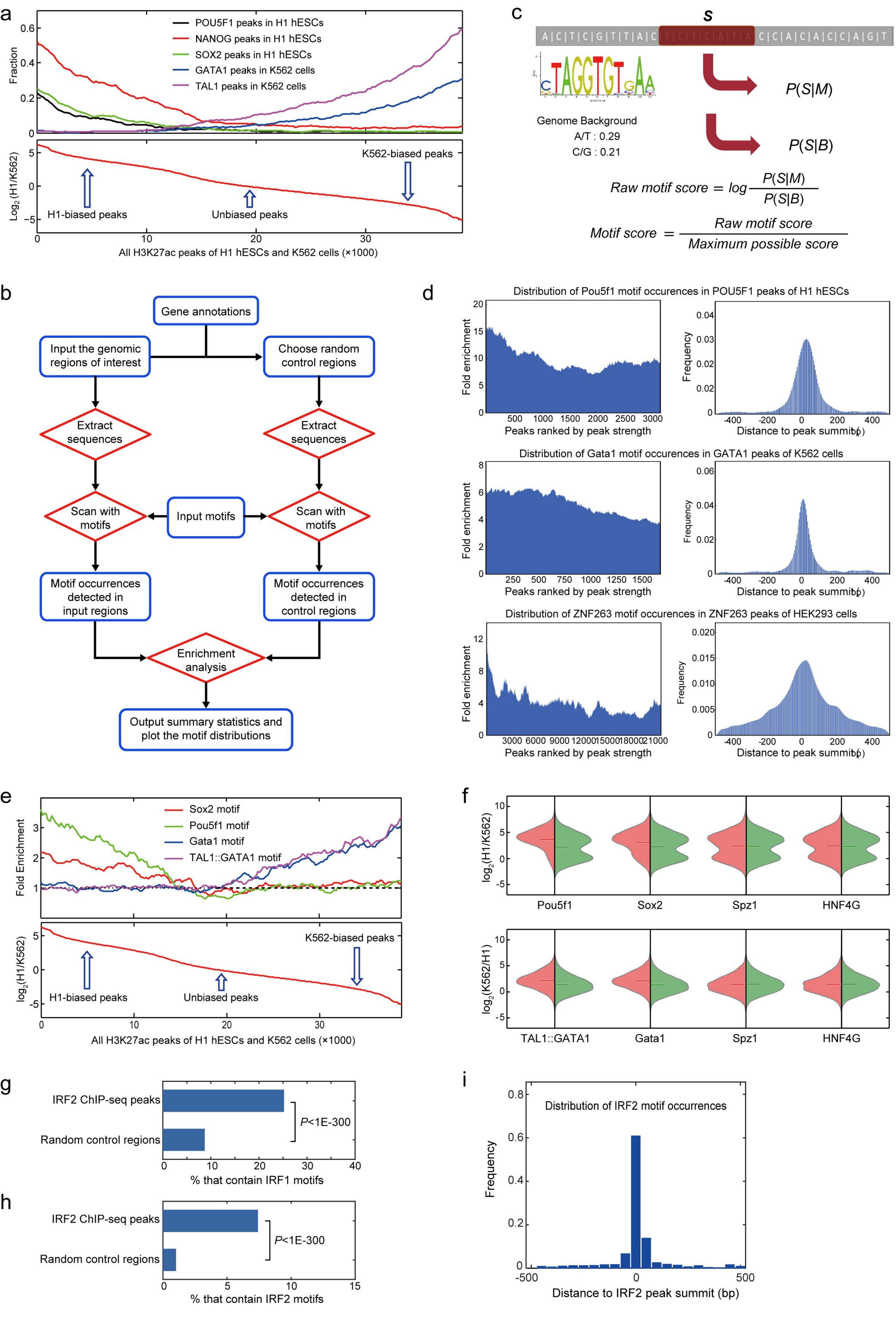
a
POU5F1 peaks in H1 hESCs
NANOG peaks in H1 hESCs
SOX2 peaks in H1 hESCs
GATA1 peaks in K562 cells
TAL1 peaks in K562 cells
Fraction
Log2 (H1/K562)
K562-biased peaks
H1-biased peaks
Unbiased peaks
All H3K27ac peaks of H1 hESCs and K562 cells (×1000)
c
s
A C T C G T T A C T C T C A T A C C A C A C C A G T
P(S|M)
Genome Background
A/T : 0.29
C/G : 0.21
P(S|B)
Raw motif score = log P(S|M) / P(S|B)
Motif score = Raw motif score / Maximum possible score
b
Gene annotations
Input the genomic regions of interest
Choose random control regions
Extract sequences
Extract sequences
Scan with motifs
Input motifs
Scan with motifs
Motif occurrences detected in input regions
Motif occurrences detected in control regions
Enrichment analysis
Output summary statistics and plot the motif distributions
d
Distribution of Pou5f1 motif occurences in POU5F1 peaks of H1 hESCs
Fold enrichment
Peaks ranked by peak strength
Frequency
Distance to peak summit (bp)
Distribution of Gata1 motif occurences in GATA1 peaks of K562 cells
Distribution of ZNF263 motif occurences in ZNF263 peaks of HEK293 cells
e
Sox2 motif
Pou5f1 motif
Gata1 motif
TAL1::GATA1 motif
Fold Enrichment
log2(H1/K562)
K562-biased peaks
H1-biased peaks
Unbiased peaks
All H3K27ac peaks of H1 hESCs and K562 cells (×1000)
f
log2(H1/K562)
Pou5f1
Sox2
Spz1
HNF4G
log2(K562/H1)
TAL1::GATA1
Gata1
Spz1
HNF4G
g
IRF2 ChIP-seq peaks
Random control regions
P<1E-300
% that contain IRF1 motifs
h
IRF2 ChIP-seq peaks
Random control regions
P<1E-300
% that contain IRF2 motifs
i
Distribution of IRF2 motif occurrences
Frequency
Distance to IRF2 peak summit (bp)

Figure S1

Supplementary Figure S1 The workflow of Motif-Scan toolkit embedded in MAmotif. (a) Fraction of H3K27ac ChIP-seq peaks of H1 hESCs and K562 cells that overlap with the ChIP-seq peaks of POU5F1, SOX2 and NANOG in H1 hESCs and the peaks of GATA1 and TAL1 in K562 cells. Here the H3K27ac peaks of two cell types were merged into one peak list and then ranked by their log2-ratios of ChIP-seq intensities between two cell types. A sliding window of size 1000 peaks moving from left to right was used to calculate the fraction of overlapped peaks. **(b)** The overall workflow of Motif-Scan toolkit. **(c)** Definition of the motif score between a specific DNA motif and a sequence fragment of the same length to measure its similarity with the motif. **(d)** Enrichment (left panels) and distribution (right panels) plots of the occurrence of JASPAR motifs Pou5f1, Gata1 and ZNF263 in the ChIP-seq peaks of corresponding TFs in H1 hESCs, K562 and HEK293 cells, respectively. In left panels, peaks were ranked by the peak strength (here represented by the P-values from peak calling programs), and the fold enrichment of each motif in peak regions compared to random control regions was calculated using a sliding window of size 500 peaks. Right panels show the distribution of detected motif occurrences in the peak regions relative to the summit of each peak. **(e)** Fold enrichment of JASPAR motifs Pou5f1, Sox2, Gata1 and TAL1::GATA1 in the H3K27ac peaks of H1 and K562 cells. Here the H3K27ac peaks of two cell types were ranked by the log2-ratios of ChIP-seq intensities between two cell types and then the relative fold enrichment was calculated using a sliding window of size 1000 peaks. **(f)** Violin plots to show the distribution of the log2-ratios of H3K27ac ChIP-seq intensities between H1 hESCs and K562 cells over the H3K27ac peaks of H1 hESCs (upper panels) and K562 cells (lower panels). In each plot, the H3K27ac peaks were first divided into two groups: peaks that contain (red) or don't contain (green) any occurrence of the corresponding motif, and the distribution was drew from each peak group separately. Horizontal bars represent the average log2-ratio of each group of peaks. **(g-h)** Fractions of IRF2 ChIP-seq peaks of adult proEs and the corresponding random control regions that are detected to contain IRF1 **(g)** and IRF2 **(h)** motifs in their sequences by Motif-Scan. Here

the *P*-values were calculated by two-tailed Fisher's exact test using hypergeometric distribution to check whether the two fractions are significantly different. **(i)** Distribution of IRF2 motifs in the IRF2 peaks.

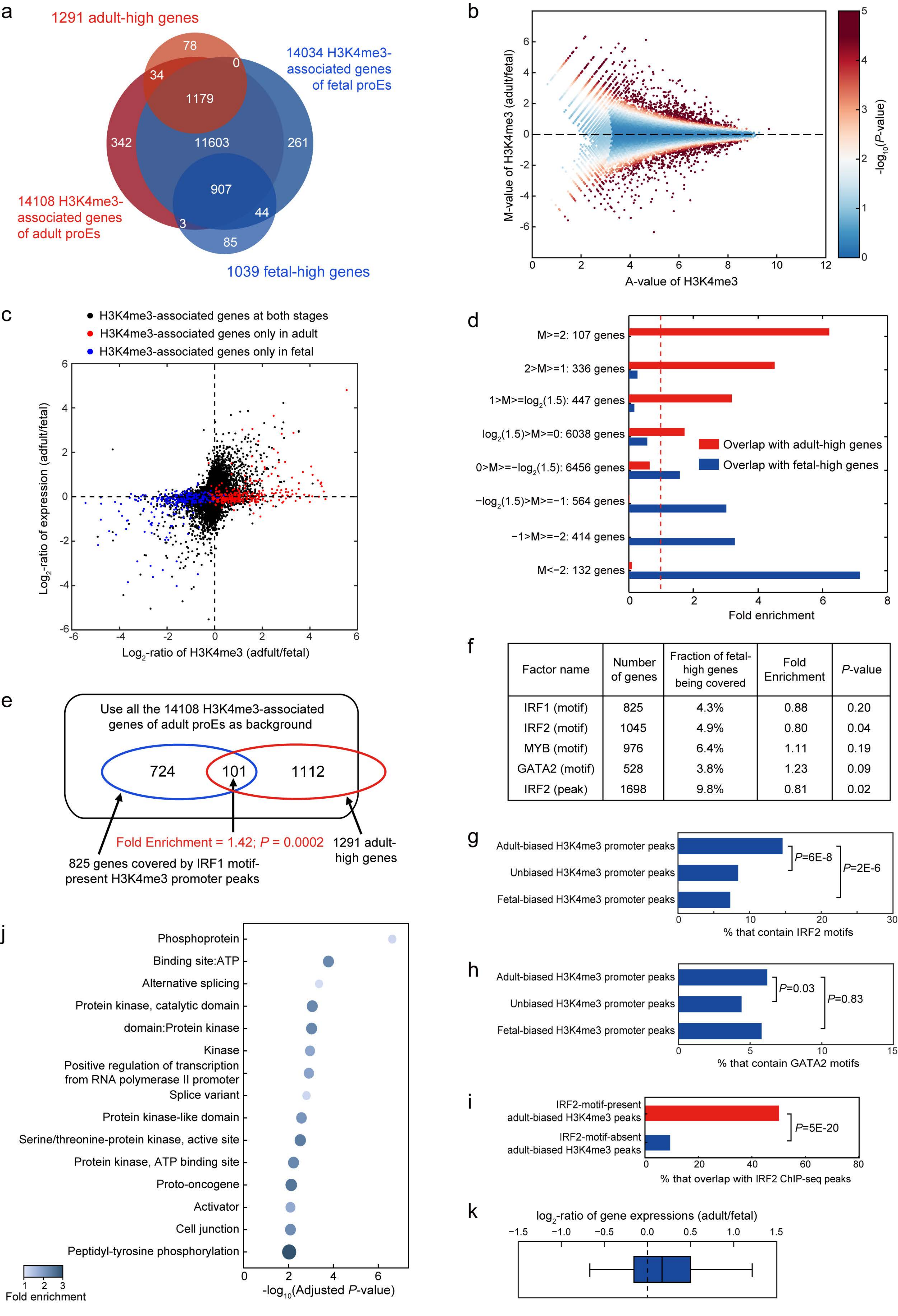

Factor name	Number of genes	Fraction of fetal-high genes being covered	Fold Enrichment	*P*-value
IRF1 (motif)	825	4.3%	0.88	0.20
IRF2 (motif)	1045	4.9%	0.80	0.04
MYB (motif)	976	6.4%	1.11	0.19
GATA2 (motif)	528	3.8%	1.23	0.09
IRF2 (peak)	1698	9.8%	0.81	0.02

Figure S2

Supplementary Figure S2 The change of H3K4me3 levels at gene promoters between adult and fetal proEs is correlated with the change of gene expression levels. (a) Venn diagram showing the overlap between the H3K4me3-associated genes of adult and fetal proEs and genes differentially expressed between adult and fetal proEs. Here the adult-high genes were defined as genes more highly expressed in adult proEs than in fetal proEs, and vice versa for the fetal-high genes. **(b)** Traditional MA plot to visualize the quantitative comparison of H3K4me3 ChIP-seq data between adult and fetal proEs using MAnorm. Here M value is defined as the log_2-ratio of normalized ChIP-seq read densities at each peak (calculated as adult/fetal), while A value is the mean log2-transformed ChIP-seq read densities at this peak, and the *P*-value is calculated by MAnorm to represent the significance of the ChIP-seq intensity change detected at each peak. **(c)** Scatter plot of the log2-ratios of gene expression levels between adult and fetal proEs versus the log2-ratios of H3K4me3 ChIP-seq intensities over all the H3K4me3-associated genes of adult and fetal proEs. Here the genes labeled as H3K4me3-associated genes at both adult and fetal stages and those labeled as H3K4me3-associated genes only in adult or fetal proEs were plotted in different colors, and the log2-ratio of ChIP-seq intensities of the H3K4me3 peak located at each gene's promoter is assigned to it. **(d)** Fold enrichment of adult/fetal-high genes in the H3K4me3-associated genes of adult and fetal proEs. Here the H3K4me3-associated genes were grouped by their log2-ratios of H3K4me3 levels between adult and fetal proEs. **(e)** The overlap between adult-high genes (genes more highly expressed in adult proEs than fetal proEs) and genes covered by the H3K4me3 promoter peaks of adult proEs that contain IRF1 motifs in their sequences. Here all 14,108 H3K4me3-associated genes of adult proEs were used as background for enrichment analysis and the P-value is calculated by right-tailed Fisher's exact test (using hypergeometric distribution). **(f)** The overlap between fetal-high genes and genes covered by the H3K4me3 promoter peaks of adult proEs that contain IRF1/2, MYB, GATA2 motifs, and IRF2 ChIP-seq peaks of adult proEs, respectively. **(g-h)** Fractions of the adult/fetal-biased and unbiased

H3K4me3 peaks defined by MAnorm that contain IRF2 **(g)** and GATA2 **(h)** motifs. **(i)** Fractions of the adult-biased H3K4me3 peaks with and without the presence of IRF2 motifs that also overlap with IRF2 ChIP-seq peaks of adult proEs. Here the *P*-values shown in (g-i) were calculated by two-tailed Fisher's exact test using hypergeometric distribution. **(j)** Gene ontology (GO) enrichment analysis of IRF2 enhancer-bound genes of adult proEs (defined as genes associated with the distal active enhancers co-occupied by IRF2 ChIP-seq peaks). **(k)** Boxplot showing the expression changes of IRF2 promoter-bound immune genes between adult and fetal proEs. Here IRF2 promoter-bound immune genes were defined as IRF2 promoter-bound genes of adult proEs associated with the 6 immune and virus response related terms shown in Figure 1h.

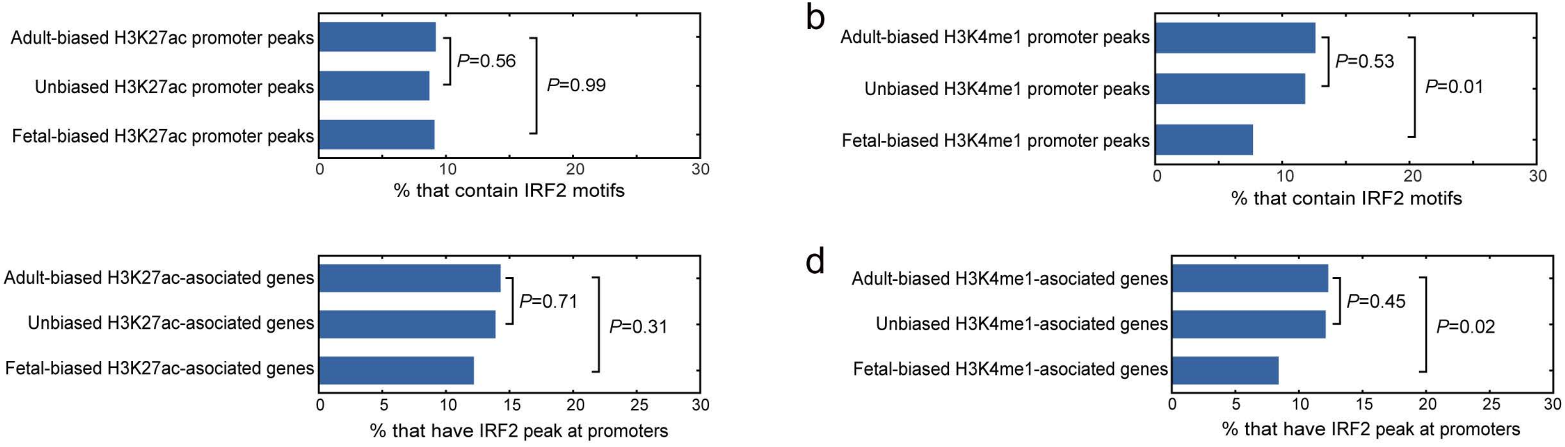

e

Gene group	Number of genes	Fraction of adult-high genes being covered	Fold Enrichment	*P*-value
IRF2 promoter-bound genes	1869	16.6%	1.91	4E-21
The second nearest genes of IRF2 promoter peaks	1497	9.2%	1.3	2E-03

f

Gene group	Number of genes	Fraction of IRF2-activated genes being covered	Fold Enrichment	*P*-value
IRF2 promoter-bound genes	1869	20.6%	2.37	3E-07
The second nearest genes of IRF2 promoter peaks	1497	7.9%	1.14	0.34

Figure S3

Supplementary Figure S3 Analysis with the ChIP-seq data of histone modifications H3K27ac and H3K4me1 in adult and fetal proEs. (a) Fractions of the adult/fetal-biased and unbiased H3K27ac peaks defined by MAnorm that contain IRF2 motif in their sequences. **(b)** Fractions of the adult/fetal-biased and unbiased H3K4me1 peaks defined by MAnorm having IRF2 motif. **(c)** Fractions of the adult/fetal-biased and unbiased H3K27ac-associated genes that have IRF2 ChIP-seq peak of adult proEs at their promoters. **(d)** Fractions of the adult/fetal-biased and unbiased H3K4me1-associated genes that have IRF2 peak of adult proEs at their promoters. Here the P-values shown in **(a-d)** were calculated by two-tailed Fisher's exact test using hypergeometric distribution. **(e-f)** Statistics of the enrichment of adult-high genes **(e)** and IRF2-activated genes **(f)** in the nearest genes (indicated as IRF2-promoter bound genes) and the second nearest genes of IRF2 ChIP-seq peaks in adult proEs.

a

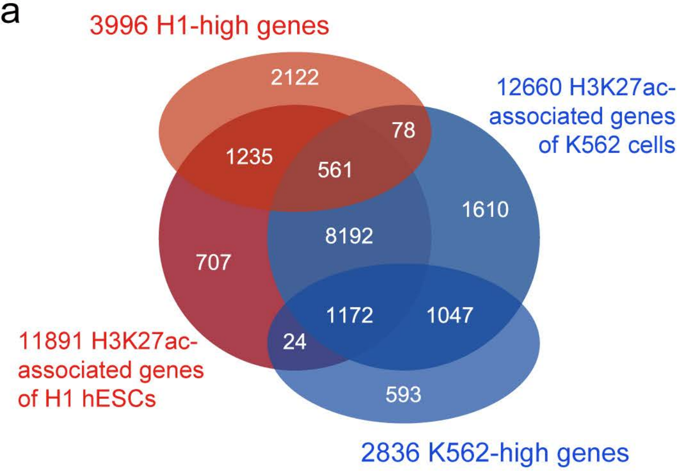

b

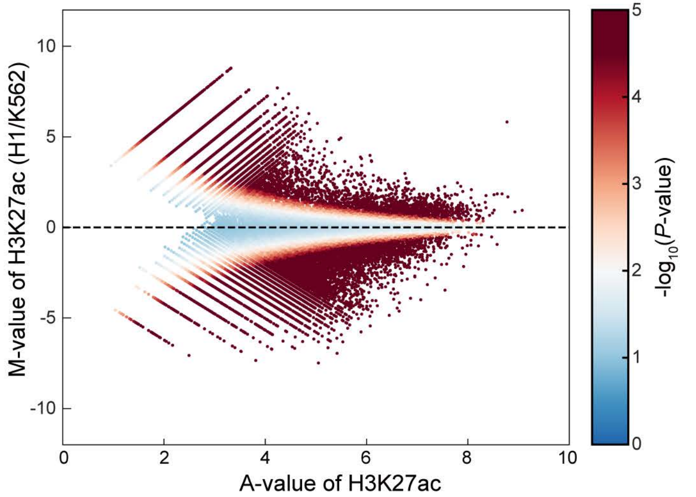

c

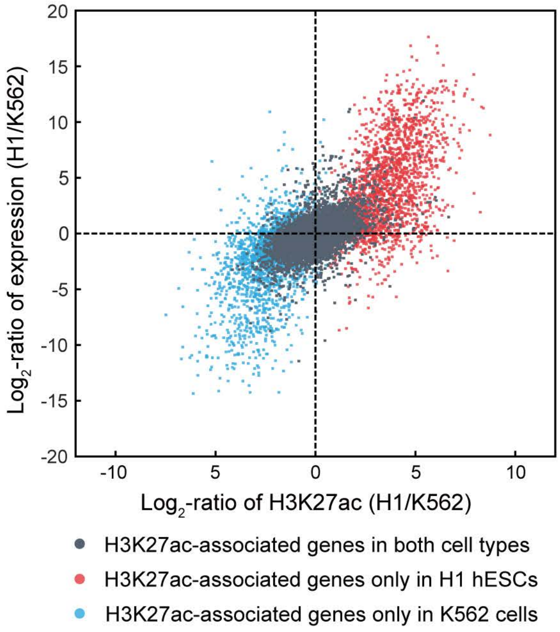

d

Motif Name	*P* (Overlap)	Rank	*P* (MAmotif)	Rank
Pou5f1	3E-141	1	3E-170	1
MZF1_1-4	5E-53	2	5E-51	4
Sox6	6E-53	3	3E-64	2
Sox3	5E-45	4	1E-57	3
Tcf3	3E-44	5	8E-50	5

Figure S4

Supplementary Figure S4 Analysis with the ChIP-seq data of histone modification H3K27ac in H1 hESCs and K562 cells. (a) Venn diagram showing the overlap between the H3K27ac-associated genes of H1 hESCs and K562 cells and genes differentially expressed between H1 hESCs and K562 cells. Here the H1-high genes were defined as genes more highly expressed in H1 hESCs than in K562 cells, and vice versa for the K562-high genes. **(b)** MA plot to visualize the quantitative comparison of H3K27ac ChIP-seq data between H1 hESCs and K562 cells using MAnorm. **(c)** Scatter plot of the log2-ratios of gene expression levels between H1 hESCs and K562 cells versus the log2-ratios of H3K27ac ChIP-seq intensities over all the H3K27ac-associated genes of H1 hESCs and K562 cells. Here the genes labeled as H3K27ac-associated genes in both H1 hESCs and K562 cells and those labeled as H3K27ac-associated genes only in H1 or K562 cells were plotted in different colors. **(d)** The top five JASPAR motifs predicted by MAmotif and traditional overlap-based approach that are significantly associated with the H1-biased H3K27ac peaks compared to K562 cells.

a

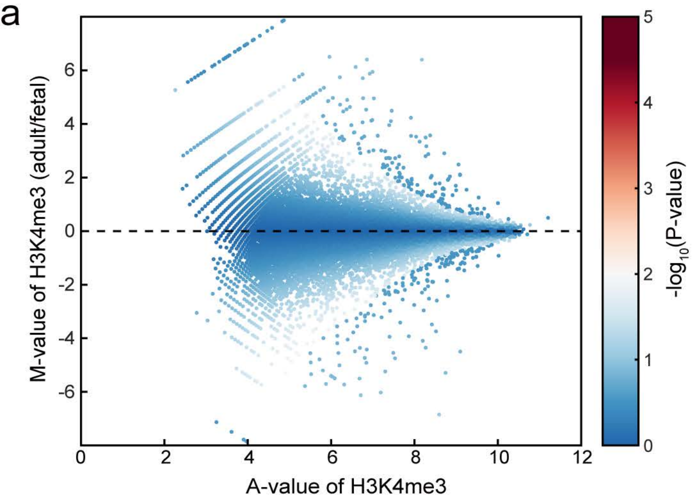

d

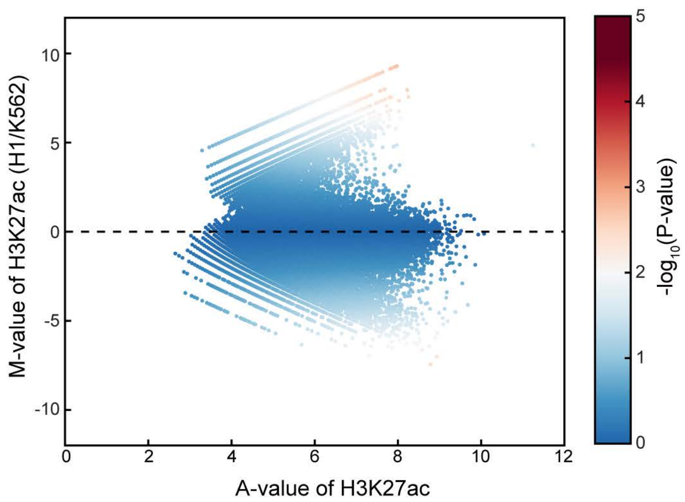

b

Comparison of H3K4me3 ChIP-seq data between adult and fetal proEs			
Using **DEseq2**		Using **MAnorm**	
Cutoffs: log_2-ratio>log_2(1.5) & P<0.01	Cutoffs: log_2-ratio<-log_2(1.5) & P<0.01	Cutoffs: log_2-ratio>log_2(1.5) & P<0.01	Cutoffs: log_2-ratio<-log_2(1.5) & P<0.01
**0** adult-biased H3K4me3 peaks	**0** fetal-biased H3K4me3 peaks	**1466** adult-biased H3K4me3 peaks	**1090** fetal-biased H3K4me3 peaks
**0** adult-biased H3K4me3 promoter peaks	**0** fetal-biased H3K4me3 promoter peaks	**505** adult-biased H3K4me3 promoter peaks	**609** fetal-biased H3K4me3 promoter peaks
Covering **0 (0.0%)** adult-high genes	Covering **0 (0.0%)** fetal-high genes	Covering **208 (16.1%)** adult-high genes	Covering **197 (19.0%)** fetal-high genes

c

Comparison of H3K4me3 ChIP-seq data between adult and fetal proEs			
Using **DEseq2**		Using **MAnorm**	
Cutoffs: log_2-ratio>log_2(1.5) & P<0.05	Cutoffs: log_2-ratio<-log_2(1.5) & P<0.05	Cutoffs: log_2-ratio>log_2(1.5) & P<0.05	Cutoffs: log_2-ratio<-log_2(1.5) & P<0.05
**211** adult-biased H3K4me3 peaks	**234** fetal-biased H3K4me3 peaks	**2571** adult-biased H3K4me3 peaks	**1841** fetal-biased H3K4me3 peaks
**86** adult-biased H3K4me3 promoter peaks	**119** fetal-biased H3K4me3 promoter peaks	**747** adult-biased H3K4me3 promoter peaks	**849** fetal-biased H3K4me3 promoter peaks
Covering **37 (2.9%)** adult-high genes	Covering **44 (4.2%)** fetal-high genes	Covering **234 (18.1%)** adult-high genes	Covering **219 (21.1%)** fetal-high genes

e

Comparison of H3K27ac ChIP-seq data between H1 hESCs and K562 cells			
Using **DEseq2**		Using **MAnorm**	
Cutoffs: log_2-ratio>1 & P<0.01	Cutoffs: log_2-ratio<-1 & P<0.01	Cutoffs: log_2-ratio>1 & P<0.01	Cutoffs: log_2-ratio<-1 & P<0.01
**2669** H1-biased H3K27ac peaks	**357** K562-biased H3K27ac peaks	**15572** H1-biased H3K27ac peaks	**13562** K562-biased H3K27ac peaks
**55** H1-biased H3K27ac promoter peaks	**3** K562-biased H3K27ac promoter peaks	**3168** H1-biased H3K27ac promoter peaks	**3521** K562-biased H3K27ac promoter peaks
Covering **54 (0.7%)** H1-high genes	Covering **3 (0.1%)** K562-high genes	Covering **1544 (38.6%)** H1-high genes	Covering **1535 (54.1%)** K562-high genes

f

ChIP-seq Samples	Number of peaks	Number of reads	Fraction of reads in the top 15,000 peaks
H3K27ac of H1 hESCs	24,624	14,894,394	11.1%
H3K27ac of K562 cells	25,000	9,150,041	44.4%
H3K4me3 of adult proEs	21,066	14,916,307	74.7%
H3K4me3 of fetal proEs	19,410	8,986,926	67.9%

Figure S5

Supplementary Figure S5 Comparison of ChIP-seq data using DEseq2. (a) Traditional MA plot to visualize the comparison of H3K4me3 ChIP-seq data between adult and fetal proEs using DEseq2. Here M value is the normalized $\log_2$-ratio of ChIP-seq read counts at each peak obtained from DEseq2 (calculated as adult/fetal), and the *P*-value is calculated by DEseq2 to represent the significance of ChIP-seq intensity change at this peak. **(b-c)** Numbers of the adult/fetal-biased H3K4me3 peaks and the adult/fetal-biased H3K4me3-associated genes defined by DEseq2 and MAnorm, respectively, using different *P*-value cutoffs. Here the fractions of the stage-biased H3K4me3-associated genes that overlap with genes differentially expressed between adult and fetal proEs were also shown. **(d)** MA plot to visualize the comparison of H3K27ac ChIP-seq data between H1 hESCs and K562 cells using DEseq2. **(e)** Numbers of the H1/K562-biased H3K27ac peaks and the H1/K562-biased H3K27ac-associated genes defined by DEseq2 and MAnorm, respectively, and the fractions of the cell type-biased H3K27ac-associated genes that overlap with genes differentially expressed between two cell types. **(f)** The fractions of aligned ChIP-seq reads covered by the top 15000 peaks of each ChIP-seq sample, to illustrate the difference between the signal-to-noise ratios of these ChIP-seq samples.
